# Management of hypertensive crisis: British and Irish Hypertension Society Position document

**DOI:** 10.1038/s41371-022-00776-9

**Published:** 2022-11-22

**Authors:** Spoorthy Kulkarni, Mark Glover, Vikas Kapil, S. M. L. Abrams, Sarah Partridge, Terry McCormack, Peter Sever, Christian Delles, Ian B. Wilkinson

**Affiliations:** 1 Department of Clinical Pharmacology and Therapeutics, Cambridge University Hospitals NHS Foundation Trust, Cambridge, CB20QQ UK; 2 Division of Therapeutics and Molecular Medicine, School of Medicine, University of Nottingham, Nottingham, NG7 2UH UK; 3 William Harvey Research Institute, Centre for Cardiovascular Medicine and Devices, Queen Mary University London, London, EC1M 6BQ UK; 4 Barts BP Centre of Excellence, Barts Heart Centre, London, EC1A 7BE UK; 5 Clinical Pharmacology and Therapeutics, Homerton Healthcare NHS Foundation Trust, London, E9 6SR UK; 6 Department of Primary Care and Public Health, Brighton and Sussex Medical School, Brighton, BN1 9PH UK; 7 Institute of Clinical and Applied Health Research, Hull York Medical School, Hull, HU6 7RX UK; 8 Imperial College School of Medicine, London, SW7 1LY UK; 9 School of Cardiovascular and Metabolic Health, University of Glasgow, Glasgow, G12 8TA UK; 10 Experimental Medicine and Immunotherapeutics, University of Cambridge, Cambridge, CB2 0QQ UK

**Keywords:** Diagnosis, Hypertension

## Abstract

Patients with hypertensive emergencies, malignant hypertension and acute severe hypertension are managed heterogeneously in clinical practice. Initiating anti-hypertensive therapy and setting BP goal in acute settings requires important considerations which differ slightly across various diagnoses and clinical contexts. This position paper by British and Irish Hypertension Society, aims to provide clinicians a framework for diagnosing, evaluating, and managing patients with hypertensive crisis, based on the critical appraisal of available evidence and expert opinion.

## Introduction

High blood pressure (BP) is the most prevalent and important modifiable risk factor for cardiovascular disease and disability, worldwide [1]. In 2015, Public Health England reported that high BP affects more than 1 in 4 adults in England. The disease burden is even larger in low- and middle-income countries (LMIC) [2–4]. There is ample, robust evidence supporting the use of antihypertensive drugs in reducing the risks of cardiovascular disease and another organ damage [5]. In contrast, acute severe elevation in BP is much less common now than in previous decades [6]. This may be attributed to more widespread screening, awareness, and better management and care models for chronic hypertension (HTN) especially in developed countries. Nevertheless, patients still present with hypertensive crises, which may be life-threatening, resulting in rapid end organ damage and/or death.

There is a lack of robust outcome data specifying BP targets, the speed of BP reduction and specific medications in patients with hypertensive crises (NICE, 2019 [7]). Management is largely based on expert opinion. Predictably, there is considerable heterogeneity and inconsistency in how severe HTN is managed in clinical practice, and often scant coverage in national and international HTN guidelines. Although the need to lower markedly elevated BP is widely appreciated, several factors need considering before initiating anti-hypertensive therapy. In this position paper, we aim to provide a framework for diagnosing, evaluating, and managing patients with hypertensive crisis, based on the available evidence.

An extensive literature search using PubMed and Embase was undertaken using keywords (see supplementary text). Relevant national, international guidelines and systematic reviews in disease-specific states were reviewed and where conflicting evidence was present individual clinical trial results were evaluated. Consensus-based positions were sought from hypertension specialists within the British and Irish Hypertension Society (BIHS).

## Definitions of elevated BP states

Various terminologies have been applied to describe severe elevation in BP, including acute severe hypertension, hypertensive urgency, hypertensive emergency, malignant hypertension (MHT), accelerated phase hypertension and hypertensive crisis. It is important to distinguish between the key terms to guide appropriate management and minimise the potential for iatrogenic harm.

### Acute severe hypertension

The definition of ‘severe’ has changed progressively in the last 70 years. Most experts would accept that a BP of >200/120 mmHg is severe, and needs urgent attention, but the degree of urgency depends on the precise circumstances. For example, a BP of 180/100 mmHg in a poorly adherent, uncontrolled chronically hypertensive patient, would not usually be considered as acute severe HTN needing immediate treatment, whereas a BP of 160/100 mmHg would be a medical (hypertensive) emergency in the context of acute end organ damage (EOD), such as preeclampsia. We prefer the term ‘acute severe hypertension’ to indicate patients with severe elevation in BP without evidence of acute and/or life-threatening EOD. Most patients presenting to emergency departments (ED) belong to this category and it is difficult to distinguish whether the rise in BP is acute or chronic especially if BP has not been measured recently. National Institute for Health and Care Excellence (NICE) guidelines (NG 136) refer to this subgroup as severe hypertension {NICE, 2019 [7]}. The term ‘hypertensive urgency’ in patients who are asymptomatic is not necessarily useful as this can lead to undue anxiety in patients and increased costs to healthcare with low associated morbidity; [8, 9] we prefer to avoid the use of this term.

### Hypertensive emergency

Hypertensive emergency is elevated BP, which when sustained over the next few hours may lead to progressive life-threatening EOD. It primarily includes conditions such as acute pulmonary oedema, hypertensive encephalopathy, acute renal failure, aortic dissection, intracerebral haemorrhage (ICH), severe preeclampsia/eclampsia, pheochromocytoma crisis and acute coronary syndrome (ACS). In several of these diagnoses, BP may be severely elevated though not necessarily. Even a moderate elevation of BP can be potentially life-threatening in certain situations, particularly if the elevation of BP is rapid. Rise in BP may not be the immediate causal factor in some emergency states like acute ischaemic stroke (AIS) and sub-arachnoid haemorrhage (SAH) but may co-exist and complicate management.

### Malignant hypertension

*MHT* is a distinct pathophysiological form of severe HTN characterised by vascular damage resulting from failure, or loss of autoregulation of blood flow, usually in patients with chronic, uncontrolled HTN. The pathophysiological hallmark is fibrinoid arteriolar necrosis in vascular tissue beds, and onion skinning of resistance vessels. Typically, diastolic blood pressure (DBP) is ≥120 mmHg, and the clinical diagnosis requires concurrent bilateral grade 3 (flame or dot-shaped haemorrhages, cotton-wool spots, hard exudates and microaneurysms) or grade 4 hypertensive retinopathy (bilateral papilloedema), as defined by Keith and Wagner classification. Whilst the prevalence of HTN and hypertensive retinopathy is higher in Afro-Caribbeans than Europeans the relationship between HTN and the prevalence of retinopathy is higher in Europeans (especially in women), and poorer in Afro-Caribbeans, especially women [10].

The prevalence of MHT is probably much higher as there’s probably an observer bias when undertaking fundoscopy. The non-availability of fundoscopy, the pupil size and the difficulty is obtaining pupillary dilator drops and/or a dark room in ED probably result in many patients with severe hypertension in whom ophthalmoscopic examination is not undertaken at all.

The most widely used classification for retinopathy changes is based on Keith, Wagner and Barker’s classification, which was first proposed in 1939. This classification has stood the test of time, especially in correlating clinical findings and prognosis. Bilateral grade 3 i.e., retinal haemorrhage, hard exudates, cotton wool spots and grade 4 i.e., optic disc swelling retinal changes essentially depict pathophysiology of severe retinal vascular permeability and are associated with poor prognosis. However, there are a few limitations to bear in mind. Correctly differentiating grade 1 (mild narrowing or sclerosis of retinal arterioles) and 2 (moderate to severe retinal arterioles, venous compressions at A-V crossing and exaggerated arterial light reflex) is subject to observer bias in everyday practice, and in some patients, these changes are probably attributable to age. The retinal changes in this classification do not describe the disease process progressively. In an acute setting, there may be no eye changes, despite other EOD showing characteristic changes in the other vascular beds such as kidney, brain, and blood vessels. Rarely some patients develop arteriolar fibrinoid necrosis appearing in the kidney before the retina, which makes the case for broadening the concept and definition of MHT.

Practically, detection of grades 1 and 2 can be used to document established chronic EOD of hypertension and grade 3 and grade 4 to document accelerated/ MHT phenotype [11].

Untreated MHT typically causes widespread vascular damage affecting a number of organs including the brain and kidneys, leading to acute renal failure, microangiopathic haemolytic anaemia (MAHA), disseminated intravascular coagulation (DIC) and hypertensive encephalopathy (complicated MHT). However, uncomplicated MHT (severe HTN with eye changes only or lone papilloedema) is not a hypertensive emergency, but a continuum of uncontrolled severe HTN, it still constitutes MHT and carries a poor prognosis without treatment. It is important that neurological imaging should be undertaken  in some patients to rule out any other aetiological causes for papilloedema [12].

MHT has been referred to as accelerated HTN variably, sometimes used to refer to severe hypertension with grade 3 hypertensive retinopathy only or without papilloedema. In this document accelerated HTN and MHT have been used interchangeably as we do not think this distinction is clinically important. MHT may recur after treatment, especially if treatment is stopped or an underlying cause continues. Eye changes usually regress with treatment, rarely without treatment.

There is significant overlap among patients with other hypertensive emergencies and MHT, i.e., complicated MHT, when MHT is accompanied by additional life-threatening EOD as described under section 2.2, it should be managed as a hypertensive emergency [13–15]. Patients with hypertensive emergencies may also be referred to as hypertension-multiorgan damage (MOD) [15].

The term ‘hypertensive crises’ has been used in the literature to describe the severity of HTN with and without organ damage. Here we are using it as an umbrella term to indicate all patients presenting with severe HTN.

## Epidemiology and risk factors for hypertensive crisis

Hypertensive crises can occur de novo in patients without any known history of HTN, and in patients who have untreated and/or uncontrolled HTN, with the latter being more common. Secondary causes of HTN including renovascular HTN, acute glomerulonephritis, renal vasculitis, drug abuse, concomitant medications, medication withdrawal and phaeochromocytoma can lead to hypertensive crisis more often than spontaneous evolution in patients with essential HTN. The estimates of hypertensive crises vary due to the different definitions used in the literature. Differing protocols with differing exclusion and inclusion criterion leads to heterogenous population cohorts being studied and reported (Table 1). Approximately 1% of patients with HTN develop an episode of hypertensive crisis in their lifetime, and acute severe HTN accounts for between 2% and 25% of all patients visiting ED [16]. Unexpectedly, the prevalence of hypertensive emergencies (defined as elevation in BP and acute end organ damage) in ED is much lower at 0.3% of all admissions [6] as noted in this meta-analysis. Hypertensive encephalopathy is even less frequent, but still accounts for ~15% of neurological, hypertensive emergencies [17]. Older age [18], male sex, background history of ongoing and uncontrolled HTN [19], chronic renal impairment and pre-existing cardiovascular disease, are some of the poor prognosis predictors of hypertensive crisis [20]. Historically, untreated MHT had an extremely high mortality rate, ~50% within 2 months of diagnosis and almost 90% by the end of 1 year [21]. 5-year survival rate has improved significantly over the years to >90% [22]. Afro-Caribbean ethnicity is noted to be a risk factor for hypertensive EOD such as hypertensive heart failure and renal failure [23, 24] confounded by higher background rates of uncontrolled HTN in the cohort [25]. However, UK data concerning ethnic differences in HTN prevalence and complications are inconsistent [19, 26].

**Table 1:** Overview of epidemiology of hypertensive crisis [6, 19, 25, 102].

Prevalence	Acute severe hypertension	Malignant hypertension	Hypertensive emergencies
	0.9% among patients presenting to emergency department (defined as hypertensive urgencies)	1–2/100,000 in Caucasian population	0.3% among patients presenting to emergency department
	11% in a predominantly African American population	7.3/100,000 in African American population	3.2% of an African American population

Nonadherence to antihypertensive medications is common, occurring in 30%-50% patients, one year after treatment initiation. It has been associated with suboptimal BP control [27], increased emergency hospital admissions [28], pseudo-resistance to medications, increased cardiovascular risks [29, 30], and ultimately increased healthcare costs.

## Assessment of a patient presenting with hypertensive crisis

**Fig. 1:**
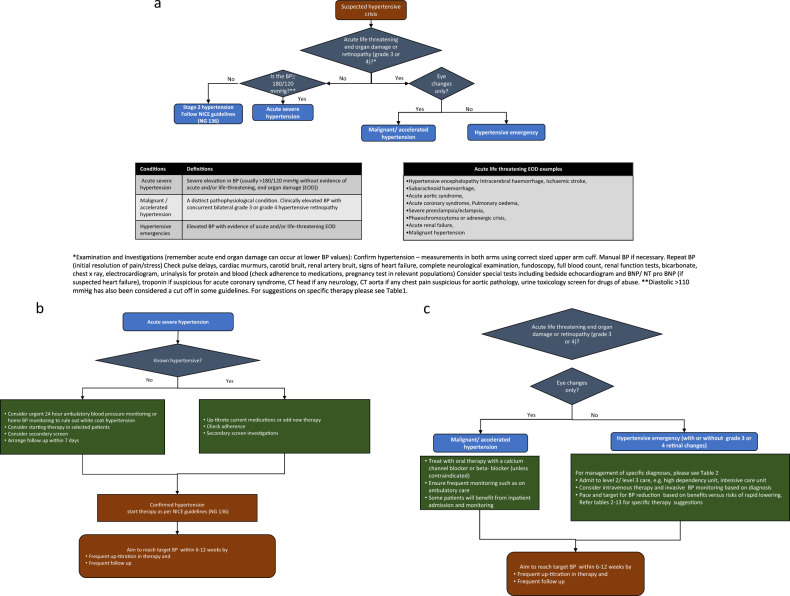
A framework for diagnosis and management of hypertensive emergencies, malignant hypertension (accelerated hypertension), and acute severe hypertension. **a** Approach and diagnosis of management of hypertensive emergencies, malignant hypertension (accelerated hypertension), and acute severe hypertension. **b** Management framework for acute severe hypertension. **c** Management framework for malignant hypertension and hypertensive emergencies.

A detailed medical history and examination (Fig. 1a–c) are the cornerstones of successful management. Background information obtained from next of kin, family/general practitioner, and medical records, complete medication review, including current and previous antihypertensive drug use and review of adherence, is essential. Use of over-the-counter medications including sympathomimetic drugs (such as ephedrine), non-steroidal anti-inflammatory drugs, chemotherapy (especially vascular endothelial growth inhibitors), oral contraceptives, herbal compounds such as liquorice use or St John’s wort, and illicit drugs such as cocaine. A complete system review to evaluate ongoing symptoms suggestive of EOD or underlying secondary causes, such as headaches, shortness of breath, palpitations and blurred vision should be undertaken. Complete examination, including BP measurement in both arms, peripheral pulsation including presence of radio-femoral delay should be undertaken. As noted above, retinal changes are key to diagnosis of MHT and hence fundoscopic examination should be performed in all patients presenting with severely increased BP with the aid of slit lamp examination and pupillary mydriasis if necessary. It is important to consider pregnancy and specifically exclude this in women in the childbearing age group with a urine pregnancy test. A bedside urine dipstick for blood and protein is important especially if nephropathy may be causal or a secondary result of HTN. However, if a life-threatening condition is suspected management should be commenced concomitantly.

Laboratory studies should include assessment of EOD, potential secondary HTN, and other cardiovascular risk factors. Assessments of renal function, electrolytes, bicarbonate, glucose, HbA1C, lipids, full blood count and clotting (looking for a microangiopathic haemolytic anaemia (MAHA) or disseminated intravascular coagulation (DIC)), bedside urine microscopy, urine albumin/creatinine ratio and thyroid function tests should be undertaken. Plasma metanephrines and/or urine metanephrines can be undertaken if phaeochromocytoma is suspected clinically. Tests for primary hyperaldosteronism (aldosterone and renin) may be helpful prior to initiating medications which may otherwise complicate interpretation. Tests for Cushing’s syndrome may be undertaken if clinically suspected. However, the testing for a secondary cause may be deferred until patient is medically stabilised. In select patients it is useful to consider urinary/objective adherence screening for anti-hypertensive medications or metabolites, especially where the clinical assessment suggests the possible non-adherence.

Imaging: In patients with neurological symptoms an urgent CT or MRI brain scan is helpful in excluding stroke or space occupying lesions and may provide support for a diagnosis of MHT. Suspicion of heart failure should prompt brain natriuretic profile (BNP)/N-terminal prohormone of brain natriuretic peptide (NT-proBNP) measurement, an urgent chest X-ray and echocardiogram, and CT aorta is mandatory for suspected aortic dissection. Renal imaging is useful for patients with elevated creatinine and to exclude renovascular lesions; CT or MRI is more helpful for the latter although expert renal artery doppler is useful where available.

Interpretation of plasma renin, aldosterone and metanephrines are confounded by various physiological factors such as volume depletion, which is often a consequence of pressure natriuresis in severe BP elevation and which leads to a picture of secondary hyperaldosteronism. Medications, pain, anxiety, Cushing’s response in conditions like SAH and other coexisting conditions such as heart failure and renal failure, can also make biochemical data uninterpretable. Secondary HTN is not always accompanied by reliable signs and symptoms, and hence many HTN centres offer secondary screening to those who present with hypertensive crisis at subsequent elective outpatient follow up, even if it is not suspected clinically. Such investigation may include screening for primary hyperaldosteronism, imaging of the adrenal glands, renal arteries and kidneys, and measurement of urinary or plasma metanephrines.

## Pathophysiology-based management of hypertensive crisis

### Principles of management

The type of EOD and specific diagnosis should direct the choice of treatment, target, and pace of BP reduction. A careful consideration of the balance of benefits and risks of rapid/excessive BP reduction and worsening acute EOD is essential. Whilst the latter is often the focus of concern, the potential harm of excessive and rapid BP reduction are real and potentially life-threatening.

A central concept is the phenomenon of autoregulation of blood flow, which ensures that organs and tissues receive constant blood flow across a wide range of arterial pressures. This is critically important for high-flow, low-resistance organs such as the brain and kidney. Normotensive people autoregulate across a relative wide range of ‘usual’ pressures approximately between 50 and 150 mmHg of mean arterial pressure (MAP) [31, 32]. In chronic HTN the cerebral autoregulation is shifted upwards, i.e., to higher MAP levels, meaning that organs cannot compensate for a sudden fall in pressure below the new lower level of autoregulation [33], and in MHT, autoregulation itself is impaired [34]. This means that lowering BP to low values, can compromise blood flow to vital organs. There are several case reports in the literature describing wide-ranging complications such as cortical blindness, stroke, and myocardial infarction, secondary to excessive, rapid reduction in BP usually by intravenous (IV) therapy [35, 36] or short-acting medications such as ‘sublingual’ nifedipine [37]. This risk can be mitigated by slower, stepwise reductions in BP beyond the lower limits of autoregulation, balanced by continuous re-assessment of the clinical impact of lowering BP reduction to ‘safe’ levels.

The exact pathophysiology leading to the severe and rapid elevation of BP is poorly understood. The release of humoral vasoconstrictors leading to endothelial damage has been implicated. This vascular ischaemic state further leads to increased systemic vascular resistance, leading to pressure natriuresis which results in further activation of RAAS. This vicious circle culminates in the initiation and perpetuation of the vascular injury in the end organs. The excessively overactive RAAS system makes patients overly sensitive to administration of agents acting on the RAAS, hence angiotensin-converting enzyme inhibitors (ACEi) and angiotensin receptor blocker (ARB) are generally best avoided in the acute phase. Rarely, this effect can also be seen with the use of β blockers, however, in most patients are considered safe enough when used at low doses.

#### Uncomplicated acute severe hypertension

NICE guidelines recommend referring patients with BP ≥ 180/120 mmHg to ED for a same-day assessment, when patients have features suggestive of acute EOD or suspicion of phaeochromocytoma, whilst recommending a repeat BP measurement reading within a week for all patients with severe HTN without EOD (acute and chronic). Similarly, both the American Heart Association (AHA)/American College of Cardiology (ACC) and the European Society of Cardiology (ESC)/European Society of HTN (ESH) guidelines state no indication for referral to the ED, nor for an immediate reduction in BP in hospital, in uncomplicated severe HTN [38–44].

During the treatment of HTN-naïve patients, it is important to ensure that confounding factors like pain, anxiety and distress are identified and addressed while assessing for acute EOD. Ambulatory BP monitoring, and/or home BP monitoring may be appropriate for some patients to investigate the persistence of elevated BP whereas in practice, close ambulatory/inpatient observation, and starting oral therapy are undertaken for many patients, particularly in a subgroup of patients such as those who do not have access to follow-up, access to home BP monitoring, pregnancy, or associated co-morbidities that may contribute to subsequent deterioration. If there is a strong suspicion of underlying nonadherence, biochemical testing, and/or directly observed therapy may be appropriate.

In patients with severe HTN without life-threatening EOD, BP should be reduced over days to weeks, rather than hours, and managed with oral therapy rather than IV (Fig. 1a, b, Tables 2 and  3).

**Table 2:** Pharmacotherapy considerations and choices for hypertensive crises states.

Hypertensive states and suggested plan of action	Pharmacotherapy choices
Acute severe hypertension Confirm hypertension, re-assess in 7 days, undertake secondary screen if applicable. Confirm adherence in known hypertension. Aim to achieve target BP in 6–12 weeks	Confirmed new hypertension: angiotensin-converting enzyme inhibitor/angiotensin receptor blocker or calcium channel blocker as per NG 136 Pre-existing hypertension: increase doses of current medications, consider adding medications as per NG 136
Malignant hypertension/accelerated hypertension (eye changes only) Start oral therapy, monitor response, arrange frequent follow up Aim to achieve target BP in days to weeks (preferably by 6 weeks, at the least by 12 weeks)	Oral atenolol 25 mg OR Oral amlodipine 5 mg/Or oral long acting nifedipine 30 mg^a^
Hypertensive encephalopathy Invasive BP monitoring Reduce mean arterial pressure (MAP) by no more than 20–25% over few hours and/or Reduce diastolic BP (DBP) to 100–110 mmHg	IV labetalol bolus 0.25–0.5 mg/kg (usually 50 mg bolus given 1–2 min, repeated within 5 min if necessary, not exceeding 200 mg), or can be administered as 15–20 mg/h continuous infusion, adjusting every 15 mins OR IV nicardipine 3–5 mg/h for 15 min, adjust with increments of 0.5 or 1 mg every 15 min, not exceeding 15 mg/h overall
Intracerebral haemorrhage Invasive BP monitoring Reduce systolic BP (SBP) by 20–25% BP targets in the range of 140–180 mmHg SBP and 90–110 mmHg DBP	IV labetalol bolus 0.25–0.5 mg/kg (usually 50 mg bolus given over 1–2 min, repeated within 5 min if necessary, not exceeding 200 mg), or can be administered as 15–20 mg/hr continuous infusion, adjusting every 15 min OR IV nicardipine 3–5 mg/h for 15 min, adjust with increments of 0.5 or 1 mg every 15 mins, not exceeding 15 mg/h overall
Ischaemic stroke Reduce BP only if specific criteria met **δ**. Reduce MAP by no more than 15% in patients with BP ≥ 220/120 mmHg	IV labetalol bolus 0.25–0.5 mg/kg (usually 50 mg bolus given over a minute, repeated within 5 min if necessary, not exceeding 200 mg), or can be administered as 15–20 mg/hr continuous infusion, adjusting every 15 min OR IV nicardipine 3-5 mg/h for 15 min, adjust with increments of 0.5 or 1 mg every 15 mins, not exceeding 15 mg/h overall
Subarachnoid haemorrhage Pain control is essential Weigh benefits and risks of reduction in BP considering cerebral vasospasm and hypoperfusion Target SBP range 140–180 mmHg	Nimodipine 60 mg orally, nasogastric, or gastric tube every 4 h, usually started within 96 h of onset of subarachnoid haemorrhage and continued for 21 days. Oral therapy preferred over other routes.
Acute aortic syndromes Pain, and anxiety control is crucial Invasive BP monitoring Reduce HR to <60 bpm, and Reduce SBP to 120 mmHg while monitoring for adequate perfusion through the false lumen	IV labetalol bolus 0.25–0.5 mg/kg (usually 50 mg bolus given over 1–2 min, repeated within 5 min if necessary, not exceeding 200 mg), or can be administered as 15–20 mg/h continuous infusion, adjusting every 15 min OR IV nicardipine 3–5 mg/h for 15 min, adjust with increments of 0.5 or 1 mg every 15 mins, not exceeding 15 mg/h overall OR IV nitroprusside initially started at 0.5–1.5 mcg/kg/min increased every 5 min by 0.5 mcg/kg/min if necessary, usual maintenance dose 10–200 mcg/min.
Acute coronary syndrome Prioritise revascularisation treatment strategy, pain and anxiety management Reduce SBP/ MAP by no more than 20–25% and avoid DBP < 70 mmHg	IV Glyceryl Trinitrate (GTN) started at 15–20 mcg/min, can increase/decrease by 5–10 mcg/min every 15–30 min, overall dose adjustment as per individual needs are met. OR IV labetalol 20 mg/h infusion, adjust every 15 min, can be administered in intermittent boluses
Left ventricular failure/Pulmonary oedema Reduce SBP/ MAP by not more than 20–25%	IV GTN 5-200 mcg/min (starting doses can start from 20–25 mcg/min), can increase/decrease by 10–15 mcg/min subsequently until required effect achieved. IV furosemide 40–80 mg (0.5–2 mg/kg) for relief of congestive symptoms (can be administered as infusion or IV boluses, subsequently oral)
Severe pre-eclampsia/eclampsia Reduce to target BP of pre-pregnancy level or ≤140/90 mm Hg in hypertension-naïve patients, reduce SBP by not more than 20% MAP in patients with very high BP values (>200/120 mmHg). Consider benefits and risks of delivery versus expectant management (NG133) Monitor maternal and foetal parameters closely.	IV magnesium, loading dose 4 g over 5–15 min followed by infusion of 1 g/h for 24 h, further dose of 2–4 g given over 5–15 min if recurrent seizures. Labetalol 200 mg oral stat or slow IV bolus injection 50 mg (over 1–2 min) which can be repeated. If IV infusion is required, at ~20–40 mg/h, up titrate as necessary. Consider addition of oral long acting nifedipine. Consider IV hydralazine slow IV injection 5–10 mg which maybe repeated or IV infusion 200–300 mcg/min: usual maintenance 50–150 mcg/min. monitor for fluid and volume status.
Phaeochromocytoma/ adrenergic crisis Maintain adequate hydration	Alpha blockade with phenoxybenzamine starting at 10 mg/day, with volume expansion using fluid and salt. Phentolamine may be used where available. Doxazosin 1–2 mg starting doses may be a suitable alternative. Beta-blockers are employed only if tachycardia ensues

*BP* blood pressure, *MAP* mean arterial pressure, *SBP* systolic blood pressure, *DBP* diastolic blood pressure, *NG-* NICE guidelines, *EOD* end organ damage, *GTN* glyceryl trinitrate

^a^Long-acting calcium channel blocker such as Adalat La 30 mg may be considered when available. Do not use short-acting nifedipine. **δ**1. If BP is greater than >220/120 mmHg in the acute phase. Lower MAP by no more than 15% over 24 h. 2. Reduce BP to 185/110 mmHg if patient is a candidate for thrombolysis 3. Reduce BP according to the concomitant emergency state as indicated. Please consult local guidelines, when available. Specialist advise should be sought for each hypertensive state, such as in the presence of renal dysfunction seek renal specialist/team advice.

**Table 3:** BIHS position: Management of acute severe hypertension.

Hypertensive state	Acute severe hypertension
Speed of BP reduction and BP targets	Recheck blood pressure (BP) to confirm hypertension.
	A. In treatment naïve patients
	1. Ambulatory blood pressure monitoring (ABPM) /home monitoring may be appropriate prior to starting treatment. Repeat BP in 7 days.
	2. If BP elevated, initiate treatment and aim to lower BP < 160/100 mmHg in 2 weeks, aim for BP < 140/90 mmHg within 6-12 weeks
	B. In known hypertensive patients,
	1. Increase doses of current therapy or add new therapy.
	2. Consider adherence testing
Other actions	Rule out white coat effect with tests mentioned above. Ensure confounders like pain, distress concomitant medications are relieved, ensure outpatient follow up and plan for up-titration in medicine to reach target BP within 12 weeks.
Medications	Follow NICE guidelines for medication choices (NG 136)

#### Malignant hypertension/accelerated hypertension

**Fig. 2:**
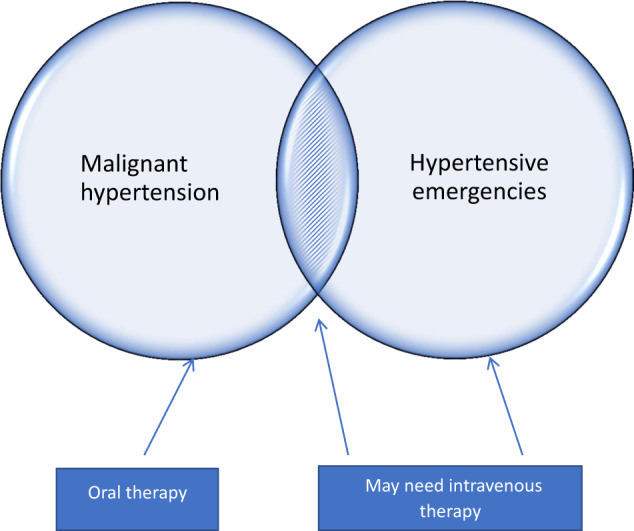
Malignant hypertension/accelerated hypertension and hypertensive emergencies, mode of therapy.

By far the safest management of uncomplicated MHT (Fig. 1c, Fig. 2) is oral therapy rather than IV therapy unless there is a co-existent emergency condition or hypertension multi-organ damage [41]. This ensures a more gradual reduction in MAP and reduces the risk of precipitous falls in blood pressure and cerebral/myocardial/renal hypoxia and necrosis. A period of observation, with arm cuff BP monitored for first few hours is usually beneficial to ensure an adequate, but not excessive, initial reduction in BP.

There is no definite evidence regarding medication of choice for patients presenting with MHT. Calcium channel blockers (CCB) such as amlodipine 5 mg or nifedipine long acting (12–24 h) 10–30 mg, or a low dose β blocker such as atenolol 25 mg may be reasonable first choices if suspicion of an underlying phaeochromocytoma is low. It is crucial, *not* to use short acting formulation nifedipine. The prescriber should be aware that CCBs with a longer intrinsic half-life (e.g., amlodipine) do take longer to achieve steady state, and might take longer to reduce BP and temptations to prescribe repeat doses within a few hours should be avoided unless there is evidence of further end-organ damage. α-blockers such as doxazosin have also been employed safely in MHT. Subsequently, β-blockers can be switched to first-line anti-hypertensive medications. If there is a suspicion on further associated EOD, or if oral therapy is not feasible, IV therapy such as labetalol or nicardipine can be employed with caution. Uncontrolled BP may result in activation of the RAAS, hence RAAS blocking agents are generally avoided in MHT, to avoid the risk of rapid BP lowering effect. Alternative approaches have been tried mostly in observational studies with variable effects [42].

In select patients, for example in patients of Afro-Caribbean ethnicity, thiazide-like diuretics can be considered at this stage along with CCBs (Table 2, Table 4).

**Table 4:** BIHS position: Management of malignant hypertension (MHT)/accelerated hypertension.

Hypertensive state	Malignant Hypertension (accelerated phase hypertension)
Speed of BP reduction and BP targets	1. In uncomplicated MHT (eye changes alone) BP is to be lowered within days, aiming for gradual reduction to reach target BP within weeks. For example: within 24 h lower to <200/120 mmHg, within a week <160/100 mmHg, then to <140/90 mmHg within 6–12 weeks.
	2. Patients with co-existent hypertensive emergency treatment should be based on end organ damage.
	3. Frequent follow up and up titration in medications are needed to reach target
Medications	Oral medications calcium channel blockers (CCB) such as amlodipine 5 mg or long acting nifedipine (20–30 mg), or low dose ß blocker for example atenolol 25 mg. Subsequently aim for the patient to continue first line medications

#### Hypertensive emergencies

Patients with hypertensive emergencies need immediate attention and, in most cases, the management should begin parallel with assessment.

### Where to treat?

Admission to high dependency unit or facility providing frequent BP monitoring and observation and ability to supervise IV medication is recommended, as IV antihypertensive agents are generally used to titrate doses to BP targets in most cases. Involvement of the relevant specialist teams is essential to provide comprehensive proficient management of the patient.

*An important principle is that a rapid lowering of BP can be more damaging to the patient than no change at all*. Most guidelines are based on expert opinion, or retrospective, observational non-randomised studies. ICH and severe pre-eclampsia are an exception to this, where larger randomised control trials (RCTs) have been undertaken (Supplementary Tables 1 and 2) for both target BP, and choice of drug. Multiple factors affect pooling of these results in emergency states and hence their usefulness and application in guiding local policies has been limited. Importantly RCTs conducted in hypertensive emergencies states have largely excluded patients with extremely high BP (typically > 220 mmHg SBP and/or >110 mmHg DBP). Moreover, most trials, set a BP target level/cut off, which fundamentally is flawed in principle if one considers the autoregulation theory described above.

Overall, there is an agreement in all international guidelines that the aim in the first 6–24 h is a controlled but *only partial* reduction of the MAP: no more than 20–25% and usually a fall in DBP of 10–15% or to ~110 mmHg, whilst aiming to maintain the DBP above 100 mmHg except in acute aortic syndromes. However, where there is evidence of chronic HTN, an even slower reduction in BP may be indicated to avoid rapid shifts in autoregulation. It is crucial to assess urine output, acid-base balance, renal function tests, and neurological state to ensure adequate end-organ perfusion.

### Which medications should be employed?

Overall, choice of antihypertensive agents (Fig. 1c, Tables 2–13) mainly includes labetalol (combined α and β blocker) which is commonly used as a first line [38, 40, 43] agent, especially in hypertensive encephalopathy, ICH, and aortic dissection along with vasodilators. Esmolol is an alternative intravenous short acting β blocker, though not frequently available. Nicardipine has also been considered as a second line therapy based on its use in some of the large trials in ICH. Advantages being that it can be safely used in patients with renal dysfunction, however, reflex tachycardia may ensue in some patients. Nitroprusside may be considered as a second-line agents except in raised intracranial pressure (ICP) states. Glyceryl trinitrate (GTN), a vasodilator is used in patients with raised BP in the context of volume-replete pulmonary oedema, acute aortic syndromes (after beta-blockade) and ACS. Other agents including, clevidipine (CCB) [44], urapidil (α-1 adrenergic antagonist and 5HT1-A antagonist) [45, 46], fenoldopam (peripheral dopamine-1 receptor agonist) [47] have been included in smaller trials in various conditions but are not currently available in the UK.

**Table 5:** BIHS position: Management of hypertensive encephalopathy.

Hypertensive state	Hypertensive encephalopathy
Speed of BP reduction and BP targets	Balance the risk of increasing cerebral oedema and ischaemia against the risk of rapid lowering of BP below cerebral autoregulation and subsequent infarction.
	1. Most patients will need invasive BP monitoring and IV therapy especially if BP elevated (above 160/100 mmHg).
	2. If BP elevated, reduce MAP by no more than 20–25% over several hours and/or reduce DBP to between 110 and 100 mmHg within 24 h.
	3. Subsequently transition to oral medications to reach and maintain BP within days to weeks.
Medications	Drug of choice: Initially IV labetalol (managed in a high dependency unit) monitoring with neuro-observations.
	If patient has asthma, calcium channel blockers can be used such as IV nicardipine.

**Table 6:** BIHS position: Management of blood pressure (BP) in acute intracerebral haemorrhage (ICH).

Hypertensive emergency state	Intracerebral haemorrhage
Speed of BP reduction and BP targets	1. Balance the risks of rapid and excessive BP reduction against the risk of ICH extension.
	2. The BP target and pace of reduction to be personalised based on background history, and ongoing response rather than a standard prespecified target.
	3. Appropriate targets are generally in the range of SBP 140-180 or DBP 90–110 mmHg.
	4. If BP is severely elevated (>220/120 mmHg), consider a smooth reduction in MAP no more than 20–25% over several hours (SBP should be kept ≥140 mmHg, preferably around 140–160 mmHg).
Medications	Drug of choice: Initially IV labetalol (managed in a high dependency unit) unless contraindicated, monitoring with neuro-observations and renal function. IV nicardipine may be suitable.

**Table 7:** BIHS position: Consideration for blood pressure (BP) lowering therapy in acute ischaemic stroke (AIS).

Hypertensive emergency state	Acute ischaemic stroke
Speed of BP reduction and BP targets	1. Balance a reduction in cerebral blood flow and increase in cerebral oedema versus haemorrhagic transformation of ischaemia. Routine anti-hypertensive treatment is not usually required
	2. If BP is greater than >220/120 mmHg, reduce by 10–15% MAP within 24 h.
	3. If BP ≤ 220/120 consider reducing to 185/110 mmHg if thrombolysis/thrombectomy indicated.
	4. All patients should have plans drawn for long term hypertension management (<140/90 mmHg prior to discharge or within few days is a reasonable aim).
Medications	If acute therapy indicated IV labetalol ±IV nicardipine or IV Glyceryl trinitrate (GTN). Choice of agent for long-term management as per NG 136.

**Table 8:** BIHS position: Considerations for blood pressure (BP) lowering therapy in subarachnoid haemorrhage (SAH).

Hypertensive emergency state	Subarachnoid haemorrhage
Speed of BP reduction and BP targets	Balance between progression of bleed and intracerebral vasospasm.
	1. BP control is not necessary unless other compelling indications such as a co-existing hypertensive emergency conditions, elevated risk of re-bleed, immediate intervention required.
	2. Adequate pain management is important.
	3. If BP reduction is required, it should be achieved with a titratable agent target of 140–180/90–110 mmHg may be appropriate.
Medications	Oral nimodipine may be considered in patients with SAH. In patients with concomitant hypertensive emergency state, treatment based on associated diagnoses.

**Table 9:** BIHS position: Management of hypertension in acute aortic syndrome (in particular type B).

Hypertensive emergency state	Acute aortic syndrome, mainly type B aortic dissection (type A dissection is a surgical emergency).
Speed of BP reduction and BP targets	1. Target SBP reduction to 120 mmHg and heart rate reduction to ≤60 bpm or the lowest level that allows as long as adequate vital organ perfusion is maintained.
	2. Ensure adequate analgesia (for example with morphine) and that agitation is controlled.
Medications	Labetalol or esmolol IV is preferred in the acute phase.
	IV nicardipine and or IV nitroprusside can be employed once heart rate is controlled. If ß blocker contraindicated, heart rate control can be achieved with non-dihydropyridine CCB. Oral medications subsequently added as tolerated.
	Long term management undertaken with oral antihypertensives to maintain a SBP target of ≤120 mmHg

**Table 10:** BIHS position: Considerations for blood pressure (BP) lowering therapy in acute coronary syndrome (ACS).

Hypertensive emergency state	Acute coronary syndrome
Speed of BP reduction and BP targets	1. Routine immediate reduction in BP is not recommended. Adequate analgesia and maintenance of oxygenation are first steps
	2. Prioritise revascularisation therapy over BP therapy
	3. Reduce BP as indicated by clinical scenario (example as per procedural requirements in the cardiac catheterisation laboratory) in patients with ACS. Do not reduce DBP below 70 mmHg.
	4. All patients should have plans drawn for long term hypertension management (<140/90 mmHg prior to discharge).
Medications	IV GTN and/or IV labetalol may be used. Nitroprusside to be avoided in ACS.

**Table 11:** BIHS position: Management of hypertension associated with acute pulmonary oedema.

Hypertensive emergency state	Acute pulmonary oedema
Speed of BP reduction and BP targets	1. Reduce BP not more than 25% reduction in MAP, while monitoring for hypoperfusion.
	2. Adequate analgesia and oxygenation are usually first steps.
	3. All patients should have plans drawn for long term hypertension management (<140/90 mmHg prior to discharge).
Medications	IV GTN or IV nitroprusside along with a loop diuretic such as IV furosemide. CCBs and IV labetalol are best avoided in the acute phase.

**Table 12:** BIHS position: Management of severe hypertension (with or without preeclampsia) associated with pregnancy.

Hypertensive emergency state	Antihypertensive management in severe pre-eclampsia/severe hypertension in pregnancy
Principles of treatment	Balancing the risk of development of eclampsia and acute hypertensive complications in the mother and delaying delivery to term (based on the possibility of prolonging gestation to allow the foetus more time to mature)
Pace of BP reduction and BP targets	1. As a first step reduce BP to <160/110 mmHg, then consider BP target ≤135/85 mmHg.
	2. If superimposed on chronic hypertension a target related to pre-pregnancy or booking BP may be more appropriate.
	3. If severe hypertension (>200/120 mmHg) a target of around 160/110 mmHg may be more appropriate. Avoid rapid drops in BP in this case while monitoring clinical and renal parameters.
	(Monitor maternal and foetal parameters closely throughout).
Medications	Labetalol, nifedipine, methyldopa, and hydralazine are considered safe during pregnancy. IV medications are easier to titrate to BP response and target especially in severe hypertension. All women should be offered routine post-natal follow-up to ensure that BP and proteinuria return to normal.

**Table 13:** BIHS position: Management of hypertension associated with phaeochromocytoma/adrenergic crisis.

Hypertensive emergency state	Phaeochromocytoma/ adrenergic crisis
Speed of reduction and targets	Targets specific to the patient, BP, and specific presentation.
Medications	α blockade with oral phenoxybenzamine (if unavailable doxazosin can be used) followed by β blockade if necessary. IV Phentolamine may be used in a crisis if needed.
	Phenoxybenzamine while preparing for surgery. β blockers are employed only if there’s persistence of tachycardia.
	Benzodiazepines for illicit drug-induced hypertension such as cocaine and amphetamine induced.
	Volume expansion with fluids and increased salt intake, as necessary to prevent postural hypotension and limit tachycardia.

Patients with ‘extremely high BP’ may have intravascular volume depletion due to pressure natriuresis, fluid balance should be managed carefully while avoiding volume overload. The dose of antihypertensive agents should be titrated carefully against BP response. Invasive monitoring, where used, should be correlated with non-invasive monitoring (SBP differs between the brachial and radial artery).

The aim of the next section is to summarise specific practice points in relation to each hypertensive emergency state. Management of acute kidney injury (AKI) as a standalone acute EOD has not been covered specifically here as it tends to overlap with MHT and other emergency states. Prevention of iatrogenic AKI is a key consideration in management of hypertensive emergencies and nephrologists should be involved at an early stage for patients presenting with AKI. For further details on management, refer to relevant national (NG148, NG203) [48, 49] and international guidelines [50, 51] for management of AKI, HTN with chronic kidney disease, and HTN associated with glomerular diseases.

## Specific hypertensive emergency states

### Hypertensive encephalopathy

This is one of the most serious though fortunately uncommon hypertensive emergencies. It refers to the transient neurological symptoms that accompany an acute rise in BP. The pathophysiology of hypertensive encephalopathy is synonymous with MHT/accelerated HTN, namely a rise in BP beyond the autoregulatory curve. A sudden, severe rise in BP results in autoregulatory failure leading to a rise in ICP, which in turn leads to a breakdown of the blood-brain barrier accompanied by arteriolar vasodilatation, cerebral oedema, and petechial micro-haemorrhage, followed by vasoconstriction.

Severe generalised headaches, lethargy, nausea, and vomiting are common symptoms, though not specific to hypertensive encephalopathy. Confusion, seizures, transient focal neurological symptoms are more suggestive. Visual disturbances such as visual field defects or blindness can occur. The most common aetiology remains uncontrolled HTN. Hypertensive encephalopathy may accompany MHT and is commonly associated with AKI and MAHA. It is a diagnosis of exclusion, and thus it is important to rule out ICH and AIS, which may also present with elevated BP and focal neurology. CT or MRI imaging is useful to rule out ICH in the first instance. CT may reveal cerebral oedema, but MRI is confirmatory, classically showing symmetrical, subcortical, parieto-occipital hyperintense lesions i.e., posterior reversible leukoencephalopathy syndrome (PRES). Though PRES is frequently associated with hypertensive encephalopathy and eclampsia, it may occur without HTN for example in association with cytotoxic or immunosuppressive therapy.

The aim of treatment is to reduce the mean arterial pressure by no more than 20–25% over several hours, or to reduce DBP to 100–110 mmHg (which of these is more appropriate will depend on the level of BP elevation), under constant monitoring. In most patients, clinical features resolve within a few hours with appropriate BP control. IV Labetalol is used frequently to control BP and nicardipine is an alternative [52] (Table 5).

### Intracerebral haemorrhage

ICH is associated with a 1-month mortality rate of 40% [53]. HTN is the attributable risk factor in ~65% of all patients with ICH. The rupture of a single vessel giving rise to an initial haematoma leads to ICH. This is preceded by an asymptomatic period where chronic HTN leads to multiple changes in blood vessels including smooth muscle cell proliferation, smooth muscle necrosis and resulting lipohyalinosis. Diagnosis is made by sudden onset focal neurological deficit, commonly associated with headache, nausea, vomiting and drowsiness. Confirmation of diagnosis is made by CT head.

The principles of treating BP in patients with ICH are the prevention of neurological deterioration due to re-bleed and/or expansion of bleeding while managing the risk of ischaemia from rapid lowering of BP. AHA/ACC guidelines recommend patients with BP ranging from 150 to 220 mmHg, have BP reduced to not less than 140 mmHg within 6 h [38]. ESC/ESH and European Stroke Organisation (ESO) guidelines recommend, patients with ICH presenting SBP > 180 mmHg, to reduce BP immediately to 130–180 mmHg [39, 54]. NICE guidelines recommend that in patients presenting within 6 h of ICH and a BP between 150 and 220 mmHg, reduce SBP to 140 mmHg or lower within 1 hour of starting treatment, not exceeding a 60 mmHg drop and maintain it for 7 days, unless suspected to have an underlying structural cause, GCS score < 6, early neurosurgical intervention planned or have a massive haematoma with a poor expected outcome [55](NG 128). A case-by-case management should be considered in patients with SBP greater than 220 mmHg.

Many of the above guidelines acknowledge the lack of robust evidence of these target BP on improvement on quality of life, mortality and/ or morbidity. Several large trials and meta-analysis have been undertaken to compare lower and rapid lowering of BP versus conservative/standard BP reduction [56] (supplementary Table). The largest of the trials, Intensive Blood Pressure Reduction in Acute Cerebral Haemorrhage Trial II (INTERACT II) trial (*N* = 2839), designed to compare intensive reduction of SBP (<140 mmHg within 1 h) to standard SBP reduction (<180 mmHg within 1 h) in patients with acute ICH showed no difference in the primary outcome of mortality or severe disability between the two arms. Three months post randomisation functional outcomes were, however, better in the intensive arm [57]. The Antihypertensive Treatment of Acute Cerebral Haemorrhage II (ATACH II) trial (*n* = 1000 randomised) evaluated aggressive lowering of BP within first 2 h (target SBP 110–139 mmHg) versus a control arm (target SBP 140–179 mmHg) and did not show any difference in outcomes [58] similar to other. A meta-analysis including INTERACT II and ATACH II, came to a similar conclusion i.e., no difference at 3 months in mortality or major disability between intensive and conservative targets [59]. In the meta-analysis, there was a higher proportion of patients with renal failure in the intensive treatment arm, while haematoma enlargement was associated with conservative treatment. Whilst decreasing size of haematoma may be desirable, the clinical impact on functional outcomes remains unclear [60].

Overall, current guidelines are based on the interpretation that intensive lowering (to SBP around or lower than 140 mmHg) appears as safe as less-intensive targets (to 160–180 mmHg), with a suggestion of possible benefit on the functional outcomes with lower targets, but at the expense of worsening renal function in select patients. Therefore, SBP of 140 mmHg may be an appropriate target value for many patients, but the onus falls on the treating physician to consider the applicability of these guidelines and trials’ findings to the patient presenting in ED, and that a more conservative target of 160 mmHg is equally evidence-based. It is also important to note that most RCTs excluded patients with extremely high BP (>220/120 mmHg) and in such patients more cautious BP lowering with higher target pressure may be indicated.

β blockers are commonly used as first line therapy, along with CCBs occasionally. In several of these trials nicardipine was used among the first line agents (Table 6).

### Acute ischaemic stroke

HTN is a common risk factor for acute ischaemic stroke (AIS), and thus, many patients presenting with focal neurological defects will be hypertensive. Chronic HTN leads to a multitude of changes in the vessel wall predisposing to thrombosis. AIS may further increase BP in patients with chronic HTN to a variable degree, but it is unclear whether this is compensatory to help maintain cerebral perfusion pressure (CPP), or purely detrimental. Similar to other hypertensive emergency states, a rapid large drop in BP can reduce cerebral blood flow leading to cerebral infarction or peri-haematomal ischaemia, while extremely high BP can lead to ongoing cerebral oedema, increased risk of haematoma expansion in ICH or haemorrhagic transformation. RCTs and subsequent meta-analysis show lack of benefit of reduction in BP to specified targets [56, 61, 62].

Treatment to reduce BP is recommended in specific scenarios by relevant ESC/ ESH, ESO and AHA/ACC guidelines ([38, 39, 53].

These indications include:

1. If BP is greater than >220/120 mmHg in the acute phase, lower MAP by no more than 15% over 24 h, to maintain perfusion of the penumbra.

2. Reduction to 185/110 mmHg should be considered in those who are candidates for thrombolysis and to maintain a BP < 185/110 mmHg for at least 24 h after receiving the tissue plasminogen activator (tPA) therapy. The evidence for the relative contraindication of tPA in patients with BP > 185/ 100 mmHg is weak, based on small nonrandomised pilot studies [63, 64].

3. Consider whether another emergency state/ EOD is present [65, 66].

Overall, the evidence or lack of evidence here raises a debate if AIS is truly a hypertensive emergency or should be categorised as chronic EOD associated with uncontrolled HTN albeit a serious consequence (Table 7).

### Subarachnoid haemorrhage (SAH)

Subarachnoid haemorrhage accounts for 2–7% of all strokes and is associated with significant morbidity and mortality. Mortality rate can be as high as 50–60% in the first few months with conservative treatment largely due to cerebral vasospasm and delayed cerebral ischaemia [67, 68]. HTN is considered a risk factor SAH.

Interventions with proven survival benefit for SAH include nimodipine, early aneurysm stabilisation, rapid transfer to high-volume treatment centres and greater use of endovascular services [69]. With each intervention the level of evidence for benefit is variable and there is significant heterogeneity in how SAH is managed [70]. Therapeutic agents that result in preferential dilation of cerebral vasculature, in turn reducing vasospasm and delayed ischaemia are considered beneficial.

Oral nimodipine is recommended in most patients with non-traumatic SAH, as shown in clinical trials and meta-analysis to improve neurological outcomes [71, 72]. Whether nimodipine leads to reduction in the intracerebral vasospasm remains unclear. There is little evidence delineating the role of other calcium channel blockers in the management of SAH- oral or non-oral. Inducing hypotension is associated with poor outcomes in SAH [73–75] and as such there is no evidence that reduction in BP is beneficial.

Overall, the decision to prescribe or continue anti-hypertensive therapy should be based on the clinical picture, background history of hypotension and a balance between risk of rebleed and hypotension (Table 8).

Cardiac hypertensive emergencies include aortic dissection, ACS, and heart failure, are all accompanied by an acute rise in BP with multiple confounding factors including pain and anxiety. Clinical discretion is required to differentiate if HTN is the causative factor, or a mere bystander similar to AIS and SAH described above.

### Aortic dissection

Aortic dissection is a devastating complication associated with remarkably high mortality rates in untreated patients. Genetic predisposition and/or degeneration from chronic HTN, aging and atherosclerosis are the usual predisposing factors. Surgical treatment is preferred in type A aortic dissection and in some cases of type B dissection including where there is evidence of a leak (bleeding into cavity) or impending rupture (propagation or development of saccular aneurysm) leading to compromise of blood flow to visceral organs. The Aortic multi-disciplinary team should consider all factors when planning regarding further vascular surgery. Medical management is the treatment of choice for acute uncomplicated type B and chronic aortic dissections. Management of pain and agitation is a priority. The basic principle of treatment is to reduce cyclical load on the wall of the aorta and flap by reducing heart rate to <60 bpm and lowering BP with β blockers [76]. Reduction of SBP to less than 120 mmHg (target ranging from 90 to 120 mmHg) within 1–2 h has been suggested by several international guidelines, position statements ([77–81] despite no RCT data to support it. This target may be reasonable in many patients, as long as commensurate with other organ perfusion such as cardiac, renal, and cerebral. Interestingly, hypotension defined as SBP < 90 mmHg is associated with increased mortality in both type A and type B (mortality odds 1.95 (1.08–3.52), 6.43 (2.18–18.98), respectively) [82].

The largest body of evidence for the management of dissection is the International Registry of Acute Aortic Dissection (*N* = 1301)[77, 82]. This retrospective study supported use of β blockers in patients with dissection specifically in Type A and CCBs such as nicardipine in type B dissection, however in practice β blockers are more commonly prescribed in all aortic syndromes. Non-dihydropyridine CCBs may be a useful choice in patients who have contraindications to β blocker therapy. IV labetalol or esmolol infusion (preferred due to shorter half-life) with or without morphine are usually considered first line to lower BP. Nicardipine, GTN, fenoldopam (not available in UK) are mentioned in AHA guidelines as alternatives. Agents that induce tachycardia such as nitroprusside and/or GTN are used only after β blockade [82, 83].

Long-term surveillance imaging and BP monitoring for all patients with aortic dissection is recommended with SBP maintained to 120 mmHg in patients where this is considered safe (Table 9).

### Acute coronary syndrome (ACS)

An elevated BP increases myocardial oxygen demand. Rapid and/or excessive lowering of BP might reduce the oxygen supply. Exaggerated responses to anti-hypertensive therapy frequently occurs in these patients. HTN per se does not determine the revascularisation strategy in ACS patients. However, stabilising patients to facilitate prompt coronary reperfusion is important and this may influence the pace of reduction of BP.

There is conflicting evidence relating to the impact of admission BP on patient outcomes. Similarly, the benefits of a particular BP target in the immediate period following ACS are uncertain. ESC guidelines recommend reducing SBP in patients with acute coronary events associated with HTN to <140 mmHg. In patients with elevated BP with concomitant ACS, guidelines suggest decreasing MAP by 20–25% over 1–2 h, followed by a more gradual reduction, whilst preparing for emergency intervention. In any case reduction of DBP to <70 mmHg is best avoided. AHA/ ACC guidelines propose treating patients with ACS and HTN with esmolol/ labetalol, ACEi and/or nitro-glycerine. β blockers are contraindicated if moderate to severe pulmonary oedema coexists. GTN used to relieve pain, should be avoided in patients with inferior ST elevation myocardial infarction (STEMI) and right ventricular infarction. Esmolol or labetalol are second line treatments. Appropriate β blockade helps reduce DBP and tachycardia, and thus myocardial oxygen demand. Nicardipine has been used safely in patients in whom β blockers are contraindicated. However, the exact benefit is yet to be outlined as some CCBs may be negatively inotropic and there have been concerns about increased mortality with the routine use of dihydropyridines. Sodium nitroprusside is avoided as a first-line agent as it can cause coronary steal by decrease regional blood flow and worsening myocardial ischaemia secondary to differential vasodilation. BP management should not delay emergency management of ACS itself.

ACEi/ ARB, and β blocker along with anti-platelet therapy and statins form part of the secondary prevention strategy (Table 10).

### Acute pulmonary oedema

Heart failure associated with raised BP may present as hypertensive crisis. Typically, shortness of breath, cough, orthopnoea, with compatible signs on CXR points towards heart failure. Underlying conditions like ischaemic heart disease or bilateral renal artery stenosis may predispose to heart failure.

Treatment should be directed toward the underlying cause and the associated pathophysiology. Patient may not always present with florid symptoms of pulmonary oedema. Flash pulmonary oedema, which occurs with elevated left ventricular filling pressures may be the first presentation albeit rarely.

Patients with acute hypertensive heart failure have been under-represented in acute heart failure trials [84]. The best available evidence for treatment of heart failure is for use of diuretics particularly loop diuretics such as furosemide or bumetanide. Treatment aiming for a decrease in BP (fall in MAP by 20–25%) over a few hours has been mentioned in most guidelines. AHA guidelines recommend treatment of pulmonary oedema with clevidipine, nitro-glycerine, nitroprusside or enalaprilat [38]. ESC guidelines, recommend treating BP ≥ 140/90 mmHg in patients with heart failure, to SBP < 140 mmHg [39, 40].

GTN is the most commonly used therapy in hypertensive heart failure, administered by continuous infusion. However, the evidence comes from small, retrospective, and non-randomised clinical trials [85]. The advantage is a quick reduction in preload and afterload. The use of higher doses of nitrates in patients with pulmonary oedema associated with myocardial infarction is supported by a few older studies [86, 87]. Small doses of opioids for vasodilatory and anxiolytic effect must be considered. Use of parenteral ACEi such as enalaprilat is supported by two studies particularly, one relatively large (*n* = 103) retrospective study of IV enalaprilat prescribed in the setting of acute heart failure [88] and another small (*n* = 20) double blinded RCT comparing IV enalaprilat versus placebo in patients with congestive heart failure [89]. However, IV enalaprilat is not routinely available. Nitroprusside may be an acceptable treatment strategy, while being cautious in patients with concomitant renal and liver failure. Clevidipine has been used safely in small studies [90, 91]. β blockers are contraindicated in acute pulmonary oedema (Table 11).

Both pulmonary oedema and ACS can be accompanied by an acute rise in BP with multiple confounding factors including pain and anxiety. Clinical discretion is required to differentiate if HTN is the causative factor, or a mere bystander similar to AIS and SAH.

### Pre-eclampsia and eclampsia

In the general pregnant population, the risk of preeclampsia is 3% to 5%. Amongst women with chronic HTN, 17% to 25% develop superimposed preeclampsia. Eclampsia carries an elevated risk of mortality and cardiovascular morbidity. The underlying pathophysiology seems to evolve around endothelial dysfunction, hyperperfusion, cerebral oedema leading to further vasoconstriction of cerebral vessels. Apart from raised BP, a multitude of symptoms are associated with severe preeclampsia. The most common symptoms include headaches, vomiting or visual disturbance. Hyperreflexia and clonus indicate ongoing cerebral oedema, which if remains unchecked can lead to seizures.

Screening for pregnancy should be undertaken in woman of child-bearing age presenting a symptoms and signs of raised BP. If pregnant, the gestational age helps in categorising the type of hypertensive disorder in pregnancy but does have significant overlap. Presentation with BP ≥ 160/100 mmHg along with proteinuria (>0.3 g/24 h) after 20 weeks of gestation is often defined as severe preeclampsia. It is important to recognise and treat preeclampsia and the treatment should be expeditious and administered quickly, with a goal to reduce but not normalise BP, with the aim of reducing risk of maternal stroke. The diagnosis of pre-eclampsia can be aided by measuring placental growth factor or the placental growth factor/ soluble FMS-like tyrosine kinase -1(sFLT) ratio.

American College of Obstetricians and Gynaecologists (ACOG) practice guidelines define, acute onset severe HTN associated with pregnancy with a cut off SBP ≥ 160 mmHg and/or DBP ≥ 110 mmHg [92]. The goal of treatment is to achieve a range of 140–150/90–100 mmHg. For severe preeclampsia or eclampsia, the target SBP is <140 mmHg during the first hour. ESC guidelines define SBP ≥ 170 mmHg or DBP ≥ 110 mmHg in a pregnant woman, as an emergency and BP ≥ 160/110 mmHg is considered severe HTN. A consensus position to lower BP to <160/105 mmHg is described [39, 40]. NICE guidelines (NG 133) [93] recommend a tighter target of 135/85 mmHg for all hypertensive pregnancies whilst acknowledging that the evidence base is weak. This BP target may be appropriate in many patients with preeclampsia and eclampsia where the baseline (pre-pregnancy and/or BP during the course of pregnancy) BP is near normal, however, it is difficult to apply this to other hypertensive disorders in pregnancy, namely gestational HTN, and chronic HTN, unless accompanied by superimposed preeclampsia, and/or adverse features due to associated concomitant EOD. The effects of reduction of BP in severe HTN in the context of preeclampsia is different from chronic severe HTN. There is no evidence that intensive lowering of BP in mild to moderate gestational and/or chronic HTN impacts upon pregnancy outcomes. The Control of HTN in Pregnancy (CHIPs) trial compared ‘less tight’ DBP target of 100 mmHg and ‘tight’ DBP target of <85 mmHg and found no difference in the primary outcome [94, 95]. A post hoc secondary analysis unsurprisingly showed a tight target reduced the incidence of ‘severe HTN’. This is in keeping with the Cochrane review findings of moderate evidence for BP control resulting in reduction in rates of severe HTN without impact on maternal or foetal clinical outcomes [96]. Overall, the benefit versus risk of a tight prescriptive target for reducing the risk of a ‘risk factor’ is unknown. As with RCTs undertaken in ICH, most trials undertaken in pregnant women, have excluded patients with severe HTN with SBP ≥ 220 mmHg.

For treatment of severe pre-eclampsia/eclampsia/ severe HTN in a critical care setting, magnesium sulphate infusion and anti-hypertensive agents such as oral or IV labetalol, and oral nifedipine and/or IV hydralazine, along with are recommended [93, 97] (NG133). Ultimately severe preeclampsia is treated by delivering the baby and HTN management does not have much impact on complications of preeclampsia such as HELPP (haemolysis, elevated liver enzymes, low platelet) syndrome (Table 12).

### Phaeochromocytoma and adrenergic crisis

Phaeochromocytoma may present with symptoms including HTN, paroxysmal episodes of palpitations, sweating, pallor, pounding headache, anxiety, tremulousness, feeling of impending death, nausea, vomiting, abdominal pain. Incidental identification on imaging, followed by biochemical testing is not infrequent. Patients may describe episodes or attacks that build up over a few minutes then subside over 15–60 minutes. These patients may have a normal BP at baseline or may have co-existent essential HTN. Rarely patients progress to develop other EOD leading to hypertensive emergency states for example hypertensive encephalopathy. Use of cocaine or amphetamine, or several prescription drugs (e.g., tricyclic anti-depressants) may present with a sympathetic crisis, with similar symptoms. Adrenergic crisis may be manifested by a short-lived BP rise; hence the BP can be safely reduced to the normal range within hours. The rise in BP may even resolve spontaneously in some cases by the time medical attention has been sought and medication sourced.

Management should focus on reversing the sympathetic stimulation and correcting the fluid volume contraction and resultant dehydration [98, 99]. The rarity of these sympathetic syndromes makes RCTs for treatment strategies non-existent and treatment is based on the underlying pathophysiology. Full oral α-blockade is the first line therapy for stable patients, for example phenoxybenzamine, and these agents are then continued as part of preparation for surgery. Doxazosin may be used if phenoxybenzamine is unavailable [100]. Addition of a calcium channel blocker may be beneficial in some cases. Adequate fluid replacement is essential (often IV initially) to correct intravascular volume depletion secondary to pressure natriuresis which paradoxically exacerbates the HTN. β-blockade is usually not required and should be avoided prior to adequate α blockade. However, selective, or non-selective β-blockade may be used to limit tachycardia, or prophylactically in patients with pre-existing ischaemic heart disease or dysrhythmias.

In a crisis, IV phentolamine may be useful for acute control of BP. If it is unavailable labetalol can be used rarely, though α-blockade with labetalol may be incomplete.

For illicit drug toxicity-induced HTN for example cocaine and amphetamines, IV benzodiazepines (BZD) are the first line treatment. BZDs by their action on gamma-aminobutyric acid receptors reduce agitation and prevent neurological complications such as seizures. If BP remains high despite BZDs, CCBs such as IV nicardipine, IV ß blockers such as labetalol infusion or IV GTN may be considered [101] (Table 13).

## Summary of international guidelines for hypertensive emergencies

The ESC/ESH and AHA/ACC guidelines (Table 14) [38–40] state that the aim of treatment of markedly elevated BP is to intensify/reinstitute oral anti-hypertensive drug therapy and ensure adequate follow-up with acknowledgement of the lack of a robust evidence base. There is overall general agreement that a patient with acute EOD is managed as hypertensive emergency, usually treated with IV anti-hypertensive therapy, in a high dependency/intensive care environment. There is, however, heterogeneity in outlined management of patients with acute severe HTN and the pace of reduction in BP in various specific scenarios. The guidelines mostly adopt a threshold BP of 180/110–120 mmHg to define severely elevated BP. Our opinion differs in that regard, as there cannot be a single cut off value of BP that is too low to cause EOD and the corollary that not all patients with ‘severely elevated BP’ need immediate treatment and assessment should be directed to define and treat the EOD. It is important to treat the patient and not the numbers. The targets for BP reduction and the pace of reduction defined in several hypertensive emergency states are not based on robust evidence and are mostly based on expert opinion. It must be widely appreciated that anti-hypertensive therapy should be personalised to the patient’s characteristics and the aim of treatment should be to reduce BP proportionally rather than to an arbitrary target value.

**Table 14:** Comparison of international guidelines of American Heart Association (AHA)/American College of Cardiology (ACC) and European Society of Cardiology (ESC)/European Society of Hypertension (ESH) guidelines and hypertensive emergencies position paper and National Institute for Health and Care Excellence (NICE) guidelines for management of hypertensive emergency states and current British and Irish Hypertension Society (BIHS) position.

Hypertensive crisis state	ESC/ESH European Society of Cardiology (ESC)/European Society of Hypertension (ESH) guidelines/position	American Heart Association (AHA)/American College of Cardiology (ACC) guidelines	National Institute for Health and Care Excellence (NICE) guidelines	BIHS position
Terms used	Urgencies and emergencies	Urgencies and emergencies included as crisis	Severe hypertension, accelerated hypertension also known as malignant hypertension	Acute severe hypertension, accelerated/ malignant hypertension, hypertensive emergency
Blood pressure (BP) values for diagnosis		>180/120 mmHg	≥180/120 mmHg (and often >220/120 mmHg) (NG136)	≥180/120 mmHg for acute severe hypertension, no specific cut off BP value for hypertensive emergencies
BP targets and recommendations on therapy in various hypertensive emergencies and acute severe hypertension				
Condition	ESC/ESH guidelines	AHA guidelines	NICE guidelines	BIHS position
Malignant hypertension (MHT)/ accelerated hypertension without hypertensive encephalopathy	Reduce mean arterial pressure (MAP) 20–25% over several hours. Drug of choice: labetalol or nicardipine.	Not explicitly defined		For uncomplicated MHT (eye changes only), oral medications amlodipine/ atenolol with monitoring and frequent follow up. Reach target BP (<135/85 mmHg at home) within days to weeks (6–12 weeks)
Hypertensive encephalopathy	Reduce MAP 20-25% at once with labetalol or nicardipine	Not explicitly defined however overall, for all hypertensive emergencies with a few exceptions, -reduce BP by max 25% over first hour, then to 160/100–110 mmHg over next 2–6 h then to normal over 24–48 h		Reduce MAP by no more than 20–25% over several hours and/or reduce DBP to 110 and 100 mmHg within 24 h
Ischaemic stroke	If BP > 220/120 mmHg or if considered for thrombolytic therapy and BP > 185/110 mmHg reduce MAP 15% in 1 h.	Lower SBP to <185 mmHg and DBP < 110 mmHg before initiation of IV thrombolysis. Maintain BP < 180/105 mmHg for first 24 h after IV thrombosis. If BP > 220/120 mmHg reduce MAP 15% in 24 h.	BP reduction to 185/110 mmHg considered in people who are candidates for IV thrombolysis. Anti-hypertensive treatment only if overlap with another hypertensive emergency	If BP is greater than >220/120 mmHg, reduce by 10–15% MAP within 24 h. If BP ≤ 220/120 consider reducing to 185/110 mmHg if thrombolysis/thrombectomy indicated. Aim for BP < 140/90 mmHg prior to discharge.
Intracerebral haemorrhage	If SBP > 180 mmHg, immediate reduction to 130–180 mmHg range (especially if initial BP ≥ 220 mmHg) with labetalol or nicardipine	Reducing BP to <140 mmHg can be potentially harmful	If presented within 6 h of ICH and SBP 150–220 mmHg, reduce BP rapidly. If presented beyond 6 h after stroke, consider same targets if SBP > 220 mm Hg. To consider risk of harm case by case. List of exclusions for rapid BP lowering explicitly mentioned (NG 128).	Appropriate targets are generally in the range of SBP 140–180 or DBP 90–110 mmHg. Reduction based on presenting BP values. If BP > 220/120 mmHg, consider a smooth reduction in MAP no more than 20–25% over several hours (SBP should be kept ≥140 mmHg, preferably around 140–160 mmHg).
Acute coronary syndrome (ACS), acute pulmonary oedema/hypertensive heart failure	Reduce SBP to <140 mmHg immediately on both conditions. ACS treated with GTN, labetalol. Pulmonary oedema treated with GTN or nitroprusside (with loop diuretic)	ACS treated with esmolol or labetalol, GTN, nicardipine. Pulmonary oedema treated with clevidipine, GTN or nitroprusside		For acute coronary syndrome: Routine immediate reduction in BP is not recommended. Adequate analgesia and maintenance of oxygenation are first steps. Prioritise revascularisation therapy over BP therapy. Do not reduce DBP below 70 mmHg. Aim for BP < 140/90 mmHg at the time of discharge Hypertensive heart failure: reduce MAP by not more than 25% reduction.
Acute aortic disease	Reduce SBP to <120 mmHg AND heart rate to <60 bpm with esmolol and nitroprusside or GTN or nicardipine	Reduce SBP to <120 mmHg within 20 min with esmolol or labetalol		Aim SBP reduction to 120 mmHg and heart rate reduction to ≤60 bpm. The target BP should allow adequate maintenance of vital organ perfusion and consider co-existing co-morbidities. Ensure adequate analgesia and agitation control
Severe pre-eclampsia	Reduce SBP to <160/105 mmHg in the first hour (immediate) with labetalol or nicardipine and magnesium sulphate	Rapid lowering. Reduce SBP to <140 mmHg in the first hour	Severe hypertension in pregnancy is >160/110 mmHg. Aim for BP < 135/85 mmHg	Consider BP target ≤140/90 mmHg. As a first step, reduce BP to <160/100 mmHg. If known chronic hypertension a target related to pre-pregnancy or booking BP may be more appropriate. If >200/120 mmHg a target of 160/100 mmHg may be more appropriate. Avoid rapid drops in BP and adequate maternal and foetal monitoring
Pheochromocytoma crisis/ Adrenergic crisis	Treat with phentolamine, nitroprusside, and urapidil or nicardipine used in per-operative period	Reduce SBP to <140 mmHg during the first hour. Phentolamine and labetalol are useful.		α blockade with oral phenoxybenzamine (if unavailable doxazosin can be used). IV Phentolamine if available in a crisis. Benzodiazepines for illicit drug-induced hypertension such as cocaine-induced, and amphetamine induced

*ACS* acute coronary syndrome, *BP* blood pressure, *MAP* mean arterial pressure, *SBP* systolic blood pressure, *DBP* diastolic blood pressure, *NG-* NICE guidelines, *EOD* end organ damage, *GTN* glyceryl trinitrate, *MHT* malignant hypertension, *ICH* intracerebral haemorrhage, *AIS* acute ischaemic stroke.

Figure 1a, b, and c and Table 2 provide synthesis of suggested approach and management of patients presenting with hypertensive crisis.

## Future

It is clear that there is a lack of robust clinical trial data and a lack of consensus over terminology and interpretation of the data available. Using BP levels as a threshold to determine if patients have a hypertensive emergency, and the use of terminologies like ‘hypertensive urgency’ dilute the strength of evidence. We suggest the development of established international standardised terminologies in this field.

As a core concept, the pace of reduction in hypertensive emergencies is based on CPP, and there is an unmet need for developing user-friendly non-invasive methods of assessing cerebral flow in routine clinical settings. This might help develop individualised BP targets leading to a controlled MAP and CPP management.

Less is known about underlying genetic, cultural, and environmental factors that predispose patients to hypertensive emergencies. Intrinsic heterogeneity in patients presenting with hypertensive crises and responses to therapy make RCTs difficult to design and follow through. To improve and develop an evidence base in this field prospective international observational studies either in the form of registries or trials need to be set up. These may not only help assess current treatment strategies but could also help develop biomarkers or a multivariate risk score system based on epidemiology, which in turn may help stratify patients adequately and enable personalised therapy. The set-up of a multi-national registry could also help identify areas with high prevalence rates of uncontrolled HTN and focus on regional trials to address knowledge gaps. Overall, any planned RCT should be pragmatic and relevant, and consider all the factors that are likely to affect the risks. Last but not the least, it is important to prevent nonadherence to medications right from the start i.e, at the point of diagnosis, by ensuring patients are at the centre of the shared decision-making. Throughout the patient’s journey perspectives change, and it is vital to continuously assess the factors that affect adherence both subjectively and objectively in a non-judgmental way, using the best available tools (biochemical testing is by far the most effective) within the clinical setting. Various strategies can be implemented including patient-focused solutions, using reminders/smart phone apps, regular follow-up, pill boxes and single combination pills. The ultimate goal of reducing the burden of cardiovascular disease resulting from uncontrolled hypertension is unattainable without patient partnership.

## Conclusion

Acute HTN management is heterogenous across various presentations, based on available resources and local expertise. High-quality evidence is either missing or where available does not necessarily conclusively favour specific rates of reduction in BP or specific medications. In conclusion, based on available evidence and where lacking consensus statements were made in consultation with the BIHS. The proposed set of recommendations for the management of acute hypertensive states aim to offer an evidence-based and consistent approach to the delivery of high-quality patient care.

## Summary

**What is known about this topic**

- Management of hypertensive crisis varies across presentations and is based on available resources and local expertise.
- Heterogeneous terminologies used in the literature to categorize hypertensive crisis states pose challenges to the exact assessment of epidemiology and evidence synthesis.
- High-quality evidence is either missing or where available does not conclusively favour specific management strategies.

**What does this study add**

- This paper critically reviews the available evidence base including various guidelines available for the management of hypertensive crises.
- We propose to categorize hypertensive crisis into acute severe hypertension, malignant/ accelerated hypertension, and hypertensive emergencies based on pathophysiology.
- We provide a practical framework for the diagnosis and management of hypertensive crisis based on the available evidence and expert opinion, which can be adapted to specific clinical settings.

### Supplementary information

Supplementary Text and Tables
