# Supplementary material for: Management of hypertensive crisis: British and Irish Hypertension Society Position document
Source: J Hum Hypertens. 2022 Nov 22;37(10):863–79. doi: 10.1038/s41371-022-00776-9 (PMC10539169; doi:10.1038/s41371-022-00776-9)
Supplement: Supplementary file 1 — Supplementary Text and Tables [file 41371_2022_776_MOESM1_ESM.docx]

Search key terms including MeSH terms used:

Hypertensive crisis, acute severe hypertension, high blood pressure, malignant hypertension, accelerated hypertension, hypertensive emergency, hypertensive urgency, hypertensive encephalopathy, intracerebral

haemorrhage, acute ischaemic stroke, subarachnoid haemorrhage, acute coronary syndrome, aortic dissection, hypertensive heart failure, acute pulmonary oedema, severe preeclampsia, severe hypertension in

pregnancy, secondary hypertension, drug-induced hypertension, adrenergic hypertension crisis, phaeochromocytoma, approach, treatment, management. Databases searched: MEDLINE, EMBASE, COCHRANE, NICE

evidence summaries, in addition, further hand searched on the internet. Dates: First search: 30/03/2021 (articles searched from inception), and updated on 19/12/2021. International and national guideline

updates were searched and updated again on 20/05/2022.

Table 1: Summary of relevant randomised controlled trials informing on blood pressure targets in the management of intracerebral haemorrhage and acute ischaemic stroke . (Please note: This is not an exhaustive list of clinical trials).

| Trial identifiers/name (including reference) | Emergency state | Population | Intervention | Outcome | Notes |
| --- | --- | --- | --- | --- | --- |
| The China Antihypertensive Trial in Acute Ischaemic stroke (CATIS trial) (1) | Ischaemic stroke | Aged > 22 years, stroke confirmed by CT/ MRI | BP treatment aim: lower systolic blood pressure (SBP) by 10-25% within 24 hours and BP <140/90 mmHg within 7 days versus no anti-hypertensive treatment. | No difference in the intervention and control arms in death or major disability. | Patients with blood pressure (BP) > 220/120 mmHg, other acute organ damage and patients for thrombolysis were excluded |
| Antihypertensive treatment in Acute Cerebral Haemorrhage (ATACH2)(2) | Intracerebral haemorrhage (ICH) | Patients presenting within 4.5 hours of onset of cerebral haemorrhage and SBP > 180 mm Hg | To evaluate two tiers of SBP reduction: >140 mm Hg,  and <140 mm Hg further divided on the basis of whether the BP lowering was maintained for 21-22 hours | No difference in rate of death or disability at 3 months between SBP < 140 mmHg and SBP > 140 mmHg. Higher rates of neurological deterioration within 24 hours and cardiac and renal related AEs in <140 group | This study highlights safety concerns related to intensive SBP reduction, including renal failure. |
| Intensive BP reduction in Acute Cerebral Haemorrhage trial (INTERACT2) (3) | ICH | Patients with spontaneous ICH | Intensive blood pressure lowering (target systolic BP <140 mmHg within 1 hour) as compared to guideline-recommended treatment (target systolic BP <180 mm Hg) | No change in the rate of primary outcome of death or severe disability in patients with two groups. Pre-specified secondary outcome analysis based on modified Rankin scores showed improved functional outcomes with intensive lowering of BP. | Larger trial |
| Rapid Intervention with Glyceryl trinitrate in Hypertensive stroke Trial-2 (RIGHT-2)(4) | Hyperacute stroke | Hyperacute stroke treatment initiated in ambulance | Transdermal GTN versus sham patch delivered to patients with acute stroke and BP> 120 mm Hg | No difference in primary outcome death or 7-level modified Rankin Scale | The trial included both categories: ischaemic stroke and ICH |
| Early Manipulation of Arterial Blood Pressure in Acute Ischemic Stroke (MAPAS)(5) | Acute ischaemic stroke (AIS) | Patients unsuitable for thrombolytic therapy were included in the study, irrespective of level of BP. | Patients randomised to three groups within 24 hours. Group 1: 140-160 mmHg, group 2: 161-180 mmHg, Group 3: 181-200 mmHg | No significant difference between the three groups in functional outcomes (primary outcome) Symptomatic intracranial haemorrhage more frequent in the higher SBP range was noted as part of secondary outcome analysis | Patients whose BP was lower than target, received saline bolus and/or IV norepinephrine. |
| Meta-analysis of intensive versus standard lowering of BP in intracranial haemorrhage(6-8) | Multiple randomised controlled trials included as defined by individual reviews | Intracranial haemorrhage | Intensive arm target –versus conservative arm target <180 mm Hg, BP at baseline 150-220 mm Hg. | No difference in the pooled risk ratio of 3-month death or major disability. | Based on post-hoc analysis in the trials, haematoma size reduction better in the intensive arm was noted, however, this did not translate to better functional outcomes necessarily. Higher proportion of renal failure in intensive treatment arm. |
|  |  |  |  |  |  |
|  |  |  |  |  |  |

Table 2: Trials highlighting efficacy and safety of medications used in hypertensive emergencies

| Medications | Condition | Characteristics | Outcomes | Notes |
| --- | --- | --- | --- | --- |
| IV Hydralazine versus oral Nifedipine (9) | Acute hypertension > 160/110 mm Hg in pregnancy ≥ 24 weeks of gestation | Double blind randomised controlled trial comparing intravenously administered hydralazine and oral nifedipine in pregnant women | Time taken to reduce blood pressure (BP) < 150/100 mm Hg was similar in both groups | Small study (n=60)  No serious adverse maternal/ perinatal side effects in either group |
| Nitroprusside versus urapidil (10) | Hypertensive emergencies excluding pre-eclampsia and eclampsia in emergency department | Safety and efficacy of urapidil versus nitroprusside | Urapidil is equally effective, compared to sodium nitroprusside, with less adverse events reported with Urapidil in comparison to nitroprusside. | Efficacy defined as BP measurement at 90 mins and 4 hours follow up period |
| Nicardipine versus nitroprusside(11) | Hypertensive crisis and acute pulmonary oedema treated in Emergency Departments | Compared efficacy of nicardipine and nitroprusside | No differences in BP response in the two groups | No long-term outcome data on patients |
| Labetalol versus nicardipine in acute hypertensive stroke  (12) | Hypertension management in acute stroke patients | Prospective comparison of labetalol and nicardipine | Therapeutic efficacy was superior with nicardipine versus labetalol, defined by percentage of patients achieving target BP. | A pseudo-randomised study. No difference in clinical outcomes |
| Labetalol versus hydralazine (13) | Hypertensive crisis in pregnancy (≥ 24 weeks gestation) | Compared efficacy of labetalol versus hydralazine in systolic blood pressure (SBP) ≥160 and/or diastolic blood pressure (DBP) ≥ 110 mmHg | No difference in BP control efficacy, no difference in adverse effects |  |
| Labetalol versus Hydralazine(14) | Severe hypertension in pregnancy (≥ 24 weeks gestation) | Treated if acute rise in SBP ≥160 and/or DBP≥ 110 mmHg | No difference in reaching BP goal. | Maternal palpitations significantly higher with hydralazine and neonatal bradycardia and hypotension with labetalol |
| Nifedipine retard versus Labetalol versus methyl dopa(15) | Severe hypertension in pregnancy (≥ 28 weeks gestation) | Treated if SBP ≥160 and/or DBP≥ 110 mmHg, acutely. Treated to SBP: 120-150 mmHg SBP or 70-100 mmHg within 6 hours | Nifedipine retard use resulted in a greater frequency of BP target attainment. No difference in adverse events. | No difference in outcomes between labetalol versus methyldopa |
| Control of hypertension in pregnancy study (CHIPS)(16, 17) | Comparing tight versus less tight BP control in patients with pre-eclampsia | Used labetalol and methyl dopa | No difference in rate of adverse perinatal outcomes. No difference in serious maternal complications. Methyl dopa was non-inferior to labetalol. | Despite no difference in primary outcome, the conclusion of the trial that severe pregnancy hypertension should receive ‘tight’ BP control |

.

Bibliography

1. He J, Zhang Y, Xu T, Zhao Q, Wang D, Chen CS, et al. Effects of immediate blood pressure reduction on death and major disability in patients with acute ischemic stroke: the CATIS randomized clinical trial. JAMA. 2014;311(5):479-89.

2. Qureshi AI, Palesch YY, Foster LD, Barsan WG, Goldstein JN, Hanley DF, et al. Blood Pressure-Attained Analysis of ATACH 2 Trial. Stroke. 2018;49(6):1412-8.

3. Anderson CS, Heeley E, Huang Y, Wang J, Stapf C, Delcourt C, et al. Rapid Blood-Pressure Lowering in Patients with Acute Intracerebral Hemorrhage. New England Journal of Medicine. 2013;368(25):2355-65.

4. Appleton JP, Scutt P, Dixon M, Howard H, Haywood L, Havard D, et al. Ambulance-delivered transdermal glyceryl trinitrate versus sham for ultra-acute stroke: Rationale, design and protocol for the Rapid Intervention with Glyceryl trinitrate in Hypertensive stroke Trial-2 (RIGHT-2) trial (ISRCTN26986053). International Journal of Stroke. 2017;14(2):191-206.

5. Nasi LA, Martins SCO, Gus M, Weiss G, de Almeida AG, Brondani R, et al. Early Manipulation of Arterial Blood Pressure in Acute Ischemic Stroke (MAPAS): Results of a Randomized Controlled Trial. Neurocrit Care. 2019;30(2):372-9.

6. Boulouis G, Morotti A, Goldstein JN, Charidimou A. Intensive blood pressure lowering in patients with acute intracerebral haemorrhage: clinical outcomes and haemorrhage expansion. Systematic review and meta-analysis of randomised trials. J Neurol Neurosurg Psychiatry. 2017;88(4):339-45.

7. Lattanzi S, Cagnetti C, Provinciali L, Silvestrini M. How Should We Lower Blood Pressure after Cerebral Hemorrhage? A Systematic Review and Meta-Analysis. Cerebrovascular Diseases. 2017;43(5-6):207-13.

8. Moullaali TJ, Wang X, Sandset EC, Woodhouse LJ, Law ZK, Arima H, et al. Early lowering of blood pressure after acute intracerebral haemorrhage: a systematic review and meta-analysis of individual patient data. Journal of Neurology, Neurosurgery &amp;amp; Psychiatry. 2022;93(1):6.

9. Sharma C, Soni A, Gupta A, Verma A, Verma S. Hydralazine vs nifedipine for acute hypertensive emergency in pregnancy: a randomized controlled trial. American journal of obstetrics and gynecology. 2017;217(6):687 e1- e6.

10. Hirschl MM, Binder M, Bur A, Herkner H, Mullner M, Woisetschlager C, et al. Safety and efficacy of urapidil and sodium nitroprusside in the treatment of hypertensive emergencies. Intensive Care Med. 1997;23(8):885-8.

11. Yang HJ, Kim JG, Lim YS, Ryoo E, Hyun SY, Lee G. Nicardipine versus nitroprusside infusion as antihypertensive therapy in hypertensive emergencies. J Int Med Res. 2004;32(2):118-23.

12. Liu-DeRyke X, Levy PD, Parker D, Jr., Coplin W, Rhoney DH. A prospective evaluation of labetalol versus nicardipine for blood pressure management in patients with acute stroke. Neurocrit Care. 2013;19(1):41-7.

13. Delgado De Pasquale S, Velarde R, Reyes O, De La Ossa K. Hydralazine vs labetalol for the treatment of severe hypertensive disorders of pregnancy. A randomized, controlled trial. Pregnancy hypertension. 2014;4(1):19-22.

14. Vigil-De Gracia P, Lasso M, Ruiz E, Vega-Malek JC, de Mena FT, Lopez JC, et al. Severe hypertension in pregnancy: hydralazine or labetalol. A randomized clinical trial. Eur J Obstet Gynecol Reprod Biol. 2006;128(1-2):157-62.

15. Easterling T, Mundle S, Bracken H, Parvekar S, Mool S, Magee LA, et al. Oral antihypertensive regimens (nifedipine retard, labetalol, and methyldopa) for management of severe hypertension in pregnancy: an open-label, randomised controlled trial. Lancet (London, England). 2019;394(10203):1011-21.

16. Magee LA, Rey E, Asztalos E, Hutton E, Singer J, Helewa M, et al. Management of non-severe pregnancy hypertension - A summary of the CHIPS Trial (Control of Hypertension in Pregnancy Study) research publications. Pregnancy hypertension. 2019;18(2210-7797 (Electronic)):156-62.

17. Magee LA, von Dadelszen P, Rey E, Ross S, Asztalos E, Murphy KE, et al. Less-tight versus tight control of hypertension in pregnancy. N Engl J Med. 2015;372(5):407-17.
